# Anthelmintic activity of selected ethno-medicinal plant extracts on parasitic stages of *Haemonchus contortus*

**DOI:** 10.1186/s13071-016-1458-9

**Published:** 2016-04-01

**Authors:** Rasika Kumarasingha, Sarah Preston, Tiong-Chia Yeo, Diana S. L. Lim, Chu-Lee Tu, Enzo A. Palombo, Jillian M. Shaw, Robin B. Gasser, Peter R. Boag

**Affiliations:** Development and Stem Cells Program, Monash Biomedicine Discovery Institute and Department of Biochemistry and Molecular Biology, Monash University, Melbourne, VIC 3800 Australia; Faculty of Veterinary and Agricultural Sciences, The University of Melbourne, Parkville, VIC 3010 Australia; Sarawak Biodiversity Centre (SBC), KM 20 Jalan Borneo Heights, Semengoh, Locked Bag No. 3032, 93990 Kuching, Sarawak Malaysia; Department of Chemistry and Biotechnology, Faculty of Science, Engineering and Technology, Swinburne University of Technology, Victoria, 3122 Australia; Department of Health and Medical Sciences, Faculty of Health, Arts and Design, Swinburne University of Technology, Victoria, 3122 Australia

**Keywords:** Medicinal plant extracts, Anthelmintic activity, Developmental assay, *In vitro*-assay

## Abstract

**Background:**

Parasitic roundworms (nematodes) cause substantial morbidity and mortality in livestock animals globally, and considerable productivity losses to farmers. The control of these nematodes has relied largely on the use of a limited number of anthelmintics. However, resistance to many of these these anthelmintics is now widespread, and, therefore, there is a need to find new drugs to ensure sustained and effective treatment and control into the future.

**Methods:**

Recently, we developed a screening assay to test natural, plant extracts with known inhibitory effects against the free-living worm *Caenorhabditis elegans*. Using this assay, we assessed here the effects of the extracts on motility and development of parasitic larval stages of *Haemonchus contortus*, one of the most important nematodes of small ruminants worldwide.

**Results:**

The study showed that two of five extracts from *Picria fel-terrae* Lour. have a significant inhibitory effect (at concentrations of 3–5 mg/ml) on the motility and development of *H. contortus* larvae. Although the two extracts originated from the same plant, they displayed different levels of inhibition on motility and development, which might relate to the presence of various active constituents in these extracts, or the same constituents at different concentrations in distinct parts of the plant.

**Conclusions:**

These results suggest that extracts from *P. fel-terrae* Lour. have promising anthelmintic activity and that more broadly, plant extracts are a potential rich source of anthelmintics to combat helminthic diseases.

## Background

Parasitic diseases cause major morbidity and mortality in animals globally, and considerable losses to food production. For instance, haemonchosis is one of the most significant parasitic diseases of livestock worldwide, affecting hundreds of millions of small ruminants (including sheep and goats) and causing substantial losses to the livestock industry estimated at tens of billions of dollars per annum [1, 2]. The causative agent, *Haemonchus contortus* (barber’s pole worm; Nematoda: Strongylida), feeds on blood in the stomach (abomasum) and causes gastritis, anaemia and associated complications, leading to production losses and death in severely affected animals. This nematode is transmitted orally from contaminated pasture to the host through a complex life-cycle [3]: eggs are excreted in the host faeces and hatch into first-stage larva (L1) usually within 1 day and then develop through to the second (L2) and third (L3) larval stages in about one week. Infective L3s are then ingested by the host, exsheath (xL3) and, after a histotrophic phase, develop through fourth-stage larvae (L4) to dioecious adults (within 3 weeks) in the abomasum.

Although a vaccine (Barbervax®) was recently released in Australia to support anthelmintic treatment programs against haemonchosis, the control of *H. contortus* and related nematodes relies largely on the use of anthelmintic drugs. The excessive use of such drugs has led to widespread resistance in these nematodes to most classes of anthelmintics [4–10], seriously compromising the control of parasites in many countries. Although the development of the compounds monepantel [11, 12] and derquantel (2-deoxy-paraherquamide) [13] have provided fresh hope for the development of new classes of nematocides, success in discovering new drugs has been limited.

Natural compounds from plants provide a unique opportunity in the search for new, effective and safe anthelmintics [14, 15]. In China, for example, plant-derived medicines have been used (for centuries) to treat many disease conditions in humans [16, 17] and other animals, including parasitic diseases [18–20]. It is likely that many of these natural medicines may be acting on pathways in worms that differ from targets of currently used anthelmintic drugs [21, 22] and, therefore, might be able to kill nematodes that are resistant to one or more anthelmintics. However, for the vast majority of such natural compounds, there has been limited systematic, scientific evaluation of efficacy, mode of action and identity of their active component(s) [23–25], and no plant-based anthelmintic is yet commercially available.

Recently, we tested eight extracts (PE1 to PE8) from the plants *Picria fel-terrae* Lour., *Linariantha bicolor*, *Lansium domesticum* and *Tetracera akara* for nematocidal activity against seven strains of the free-living nematode *Caenorhabditis elegans* (one wild-type and six strains with GFP-tagged stress response pathways), and characterised the stress responses caused by these extracts [26]. These plants are widely distributed throughout Asia and have been used by indigenous Malaysian healers to treat worm infections and gastrointestinal disorders in humans [26–28]. Five of the eight plant extracts (designated PE1, PE2, PE4, PE5 and PE7; Table 1), had significant nematocidal activity against both larval and adult stages of *C. elegans* [26]. The most effective extracts were from *P. fel-terrae* [26], and triggered stress response pathways that were distinct from commercially available anthelmintics (doramectin and levamisole). This study showed that using traditional knowledge of plant medicinal properties, in combination with a *C. elegans in vitro*-screen, provided a practical and economical approach to search for nematocides. Despite this progress, these plant extracts had not been tested on any parasitic nematodes.

**Table 1:** Sources of the plant extracts used in this study

Extract/origin	SBC specimen reference number	Plant species	Plant family
PE1/whole plant	A000423010301	*Picria fel-terrae* Lour.	Scrophulariaceae
PE2/leaves	A000423020301	*Picria fel-terrae* Lour.	Scrophulariaceae
PE4/roots	A001293020103	*Linariantha bicolor*	Acanthaceae
PE5/leaves	A001293030103	*Linariantha bicolor*	Acanthaceae
PE7/whole plant	A002698010103	*Lansium domesticum*	Meliaceae

The recent development of a new and inexpensive whole-organism assay for the rapid screening of chemical compounds against parasitic stages of *H. contortus* [29, 30] has provided a unique prospect to screen plant extracts for activity against one of the most important parasitic nematodes of small ruminants, as a starting point for future assessments on other nematodes. This screening assay, which relies on video-capture to measure the inhibitory properties of compounds on the motility of parasitic larval stages of *H. contortus* and subsequent, morphological assessment of larval development, was employed specifically to screen extracts PE1, PE2, PE4, PE5 and PE7 against this parasitic nematode.

## Methods

### Preparation of plant extracts for screening and assay plate preparation

Plant extracts (Table 1) were prepared by the Sarawak Biodiversity Centre (SBC), Kuching, Malaysia, as described previously [26]. Briefly, whole plants, or parts thereof, were dried, ground into a powder, extracted into 1:1 v/v dichloromethane:methanol and then concentrated using a rotary evaporator. Dried plant extracts were stored at SBC and sent to Australia upon request. Before their use, the dried plant extracts were dissolved in absolute ethanol (Merck, Australia). Serial dilutions (1–5 mg/ml) of these extracts (PE1, PE2, PE4, PE5 and PE7) were prepared in Luria Bertani medium (LB) [10 g tryptone (cat no. LP0042; Oxoid England), 5 g yeast extract (cat no. LP0042; Oxoid), 5 g NaCl (cat no. K43208004210; Merck, Denmark) in 1 l of sterile water]. LB was autoclaved and supplemented with final concentrations of 2.5 μg/ml of amphotericin, 100 IU/ml of penicillin and 100 μg/ml of streptomycin (Fungizone®, antibiotic-antimycotic; cat no. 15240–062; Gibco); this supplemented LB was designated LB*. Compounds were then dispensed in 50 μl volumes in triplicate into the wells of sterile 96-well, flat-bottom microplates (cat no. 3635; Corning 3650, Life Sciences). The anthelmintic monepantel (Zolvix®, Novartis Animal Health, Switzerland) was used at 20 μM as the positive-control compound, and LB* containing 1 % ethanol (Merck) was used as the negative-control.

### Production of *H. contortus* and storage

*Haemonchus contortus* (Haecon-5 strain) was maintained in experimental sheep as described previously [31], and in accordance with the institutional animal ethics guidelines (permit no. 1111938; The University of Melbourne). In brief, helminth-free Merino sheep (eight weeks of age) were inoculated intra-ruminally with 5,000 third-stage larvae (L3s) of *H. contortus.* Four weeks after infection, faecal samples were collected each day. L3s were produced from eggs by incubating faeces at 27 °C for one week. Then, L3s were sieved through two layers of nylon mesh (pore size: 20 μM; Rowe Scientific, Australia) to remove debris or dead larvae, and stored at 10 °C for up to three months.

### Exsheathment of L3s

L3s were exsheathed and sterilised by incubation in 0.15 % v/v sodium hypochlorite (NaClO) at 37 °C for 20 min [32]. Following this incubation, exsheathed L3s (xL3s) were washed five times in sterile physiological saline by centrifugation at 600 *x g* (5 min) at room temperature (22–24 °C). After the last wash, xL3s were suspended in LB* at a density of 300 xL3 per 50 μl.

### Screening xL3s for reduced motility upon exposure to plant extracts

We assessed the effect of plant extracts on xL3 motility, essentially as described recently [29, 30]. In brief, on 96-well plates, test compounds (at concentrations ranging from 1–5 mg/ml), the positive-control compound (monepantel) and the ethanol-control in LB* were arrayed in triplicate; six wells were used for the negative-control (LB* + 1 % ethanol). Then, 300 xL3s in 50 μl of LB* were transferred to each well of each plate (with the exception of perimeter wells) using a multi-channel pipette. The final concentration of the positive-control anthelmintic (monepantel) was 20 μM, and plant extracts (Table 1) were individually tested at 1, 2, 3, 4 and 5 mg/ml. Plates were incubated at 38 °C and 10 % v/v CO_2_. After 48 and 72 h, plates were agitated (126 rotations per min) using an orbital shaker for 30 min at 38 °C. To capture the motility of xL3s, a 10 s video recording of each well on each plate was captured using an eyepiece camera (Dino-eye, ANMO Electronic Corporation, Taiwan) attached to a stereo dissecting microscope (Olympus, Japan). After 3 min of imaging, plates were re-agitated for 5 min. The motility of xL3s was recorded in each well on each plate. Each 10 s video was processed using a custom macro in the program Image J (1.47v, imagej.nih.gov/ij) to measure larval motility, represented by the motility index (= Mi), in each well [30]. All extracts were tested individually in triplicate on three different days. Differences in motility between treated and untreated worms (negative-controls) were assessed by statistical analysis using one-way ANOVA.

### Evaluation of the effects of extracts on the development of xL3 to L4

Following the measurement of larval motility, larvae in 5 mg/ml of plant extracts were re-incubated for four more days at 38 °C and 10 % v/v CO_2_ in a humidified environment. Subsequently, worms were fixed through the addition of 50 μl of 1 % iodine, and 30 worms from each well were examined at 20x magnification to assess the development of *H. contortus* L4s (based on the presence/absence of a well-developed pharynx/mouth; cf. [29, 33]). The number of L4s was expressed as a percentage of the total worm number (*n* = 30). All compounds were tested in triplicate on three different days. To establish whether the inhibitory effect of individual plant extracts was reversible, a separate (‘recovery’) assay was conducted: xL3s incubated at 5 mg/ml plant extracts for 72 h (same conditions as above) were washed four times with 200 μl LB* to remove extracts from wells, fresh LB* (100 μl) added, and plates incubated for four more days under the same conditions. Then, worms were fixed in 1 % iodine and development to the L4 stage assessed. Differences in development between treated and untreated worms (negative-controls) were assessed by statistical analysis using one-way ANOVA.

## Results

**Fig. 1:**
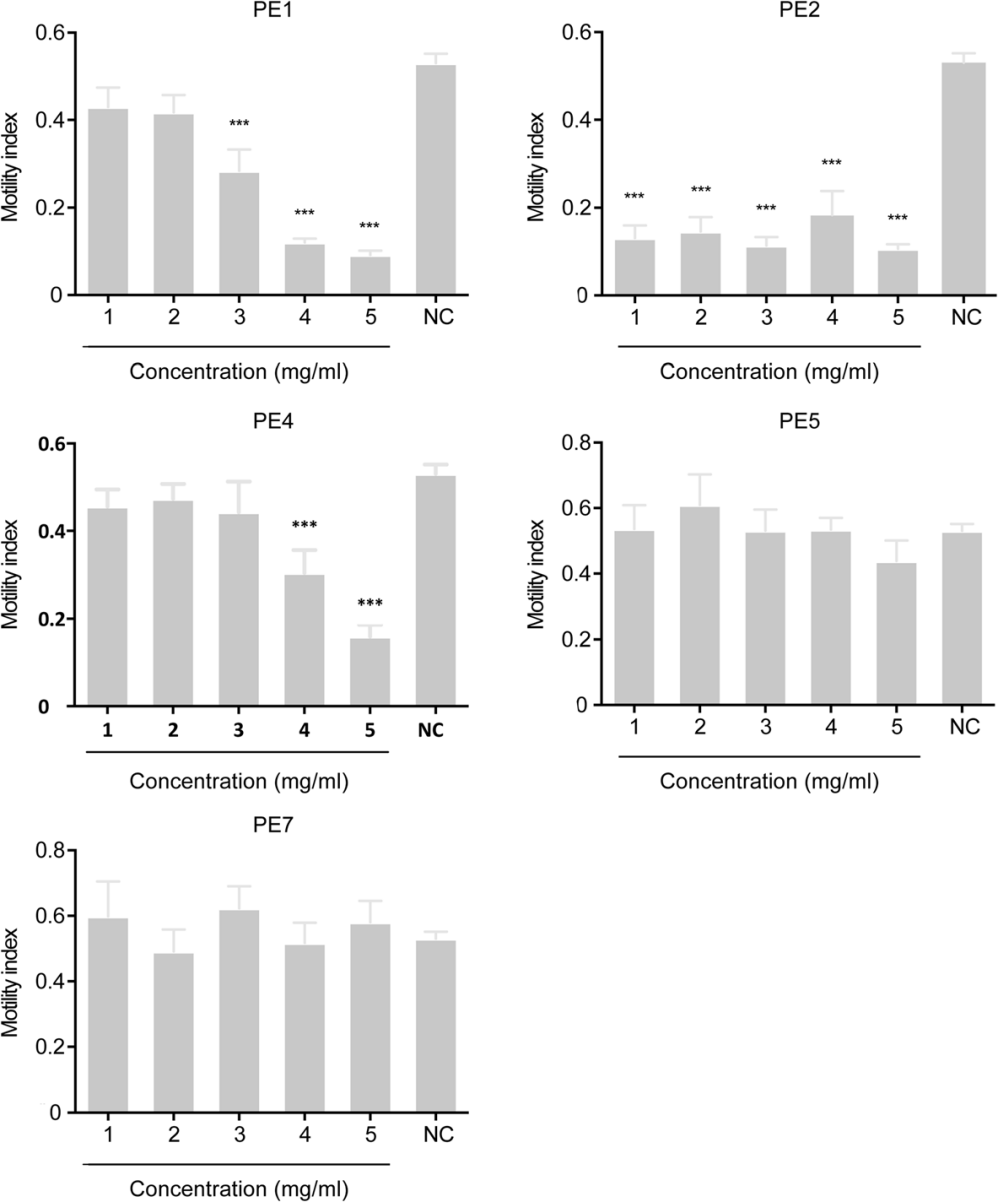
The effects of plant extracts on exsheathed third-stage larvae (xL3) of *Haemonchus contortus* after 48 h. Results were calculated from three biological and technical replicates (cf. Table 1) (300 worms per well per replicate). Error bar indicates the standard error of the mean (SEM). Negative-control (NC) represents LB* containing 1 % ethanol. ***P ≤ 0.001

**Fig. 2:**
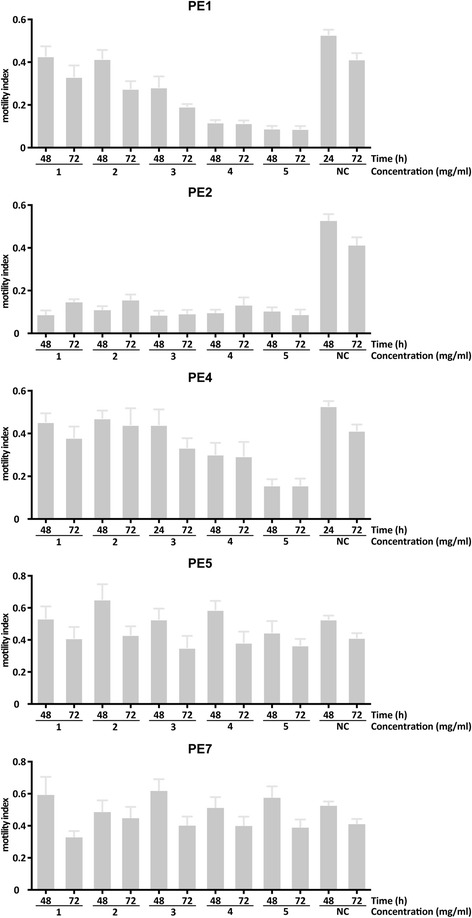
The effects of plant extracts on exsheathed third-stage larvae (xL3) of *Haemonchus contortus* after 48 h and 72 h. Results were calculated from three biological and technical replicates (cf. Table 1) (300 worms per replicate). Negative-control (NC) represents LB* containing 1 % ethanol. Error bar indicates the standard error of the mean (SEM)

To test the effects of the five plant extracts on the motility of xL3 stage of *H. contortus,* PE1, PE2, PE4, PE5 and PE7 were each assessed at five concentrations (1, 2, 3, 4 and 5 mg/ml). Both PE1 and PE4 significantly reduced xL3 motility in a dose-dependent manner with reference to the negative-control. Although PE1 did not reduce motility significantly at 1 and 2 mg/ml, it did at 3, 4 and 5 mg/ml (Fig. 1). PE4 significantly reduced xL3 motility at 4 mg/ml and 5 mg/ml, but did not affect motility at lower concentrations (Fig. 1). PE2 had a significant, adverse effect on xL3 motility at all five concentrations tested, but this effect was not dose dependent (Fig. 1). Neither PE5 nor PE7 significantly reduced xL3 motility with reference to negative-controls (Fig. 1). Although PE1, PE2 and PE4 significantly inhibited xL3 motility after 48 h (PE1: *F*_(5, 48)_ = 28.77; PE2: *F*_(5, 48)_ = 27.24; PE4: *F*_(5, 48)_ = 9.802; all *P* ≤ 0.001), these extracts did not demonstrate statistically significant, time-dependent effects on these larvae, with no increased inhibition of motility at 72 h (Fig. 2). Negative-controls showed a constant motility index (Mi) throughout all experiments.

**Fig. 3:**
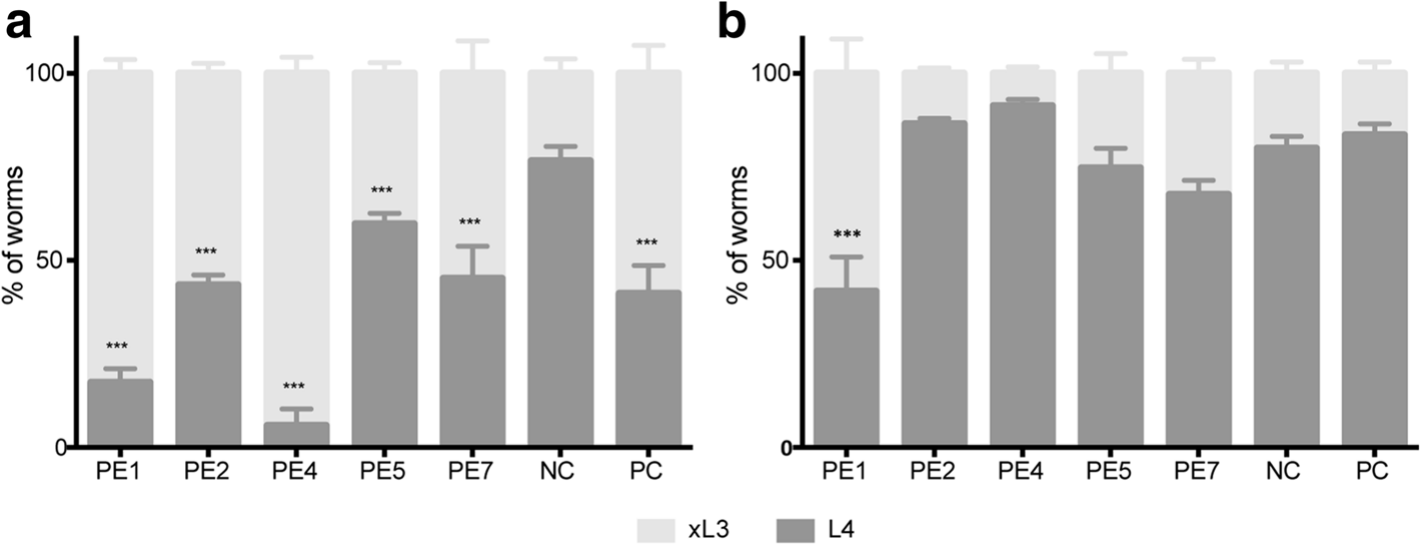
The effects of plant extracts (cf. Table 1) on the development (panel **a**) and recovery (panel **b**) of exsheathed third-stage larvae (xL3) of *Haemonchus contortus*. Monepantel (20 μM) was used as the positive-control (PC). The results were calculated from three biological replicates (300 worms per replicate). Negative-control (NC) represents LB* containing 1 % ethanol. Error bar indicates the standard error of the mean (SEM)

After seven days, the effects of individual extracts on L4 development were assessed (Fig. 3). After this time, 76.7 % of untreated xL3s developed to L4s in negative-control wells. By contrast, 17.3 % and 5.9 % of xL3s exposed to 5 mg/ml of PE1 and PE4, respectively, developed to L4s after 7 days. Although PE2, PE5, PE7 and monepantel reduced the development of xL3 to L4, this reduction was not significant (Fig. 3a). Approximately 80 % of untreated xL3s as well as xL3s exposed to extract PE2, PE4, PE5 or PE7 (5 mg/ml each) or monepantel (20 μM) developed to L4s following the addition of fresh LB*, after 72 h of incubation. By contrast, only 41.8 % of xL3s exposed to extract PE1 developed to L4s, even in the subsequent absence of PE1, after incubation for 72 h (Fig. 3b).

## Discussion

The present results show that extracts PE1 and PE2 from *P. fel-terrae* Lour. have considerable activity against the parasitic larval stages of *H. contortus in vitro*. Extract PE2 was the most effective inhibitor of xL3 motility at all concentrations tested, but with no observable dose-dependent effect. This latter extract reduced worm motility, even at the lowest concentration tested (1 mg/ml), which is consistent with its effect on *C. elegans* [26]. Although PE1 and PE2 originate from the same plant, they showed distinctive inhibitory characteristics on both xL3 motility and L4 development of *H. contortus*, which might relate to the presence of different active constituents in these extracts, or the same constituents at different concentrations in different parts of the plant. Interestingly, none of the plant extracts showed significant, time-dependent effects on the larvae at the time points tested. This finding may be due to: (i) the active constituents in plant extracts having a maximum effect on *H. contortus* within 48 h; (ii) the degradation of active constituents in the extracts during testing in the assay, such that they are no longer effective against xL3s or L4s; (iii) xL3s may have rapidly developed an acute “resistance” or used their defence mechanism to overcome some of the effect(s) of the active constituent(s), for instance, *via* a complex mechanism on the cuticle of the worm [34–36].

In larval development and recovery assays, only PE1 had an adverse impact on development from xL3 to L4. Even though both PE1 and PE4 had a remarkable, adverse effect on the development of xL3s to L4s, most PE4-treated larvae recovered and developed to L4s after the removal of the plant extract and replacement with LB*. This finding suggests that the effect of extract PE4 is reversible, whereas PE1 appears to irreversibly inhibit xL3 motility and larval development. Interestingly, a similar pattern to PE4 was observed for monepantel at a dose of 20 μM. Given that the nematodes can develop resistance rapidly (sometimes after as few as three generations; [37]), a similar situation might be the case for the active, natural compound PE4. Thus, resistance development in *H. contortus* should be evaluated*.*

The finding that three of the same five plant extracts shown previously to affect *C. elegans* [26] significantly reduced motility and development in xL3s of *H. contortus* indicates variation between the free-living and parasitic nematodes in targets and/or the pathways, which might relate to genomic differences and evolutionary distance between these worms [38–40]. However, it is also possible that the absorption of constituents from the plant extracts differs between the two worms [41], or that, from a biological/evolutionary perspective, the parasitic nematode (*H. contortus*) is perhaps more adapted to a plant extract-rich environment in the abomasum of its ruminant host compared with the soil nematode, *C. elegans*. These aspects and differences in susceptibility or efficacy need to be taken into consideration when the focus of screening is on extracts or compounds expected to have an effect on a relatively wide range of related nematodes

## Conclusion

Significant activities previously identified for PE1 and PE2 in *C. elegans in vitro* [26] were also seen in *H. contortus*. These findings suggest that extracts from *P. fel-terrae* Lour. have promising anthelmintic effects. We propose that future work should focus on attempting to fractionate extract PE1, in order to identify and characterise the constituent(s) that are active against *H. contortus*, and then to explore which biological pathways are affected by these components/fractions.

## References

[1] Roeber F, Jex AR, Gasser RB (2013). Next-generation molecular-diagnostic tools for gastrointestinal nematodes of livestock, with an emphasis on small ruminants. A turning point?. Adv Parasitol.

[2] Waller PJ, Chandrawathani P (2005). *Haemonchus contortus*: parasite problem No. 1 from tropics - Polar Circle. Problems and prospects for control based on epidemiology. Trop Biomed.

[3] Veglia F (1915). The anatomy and life-history of the *Haemonchus contortus* (Rud.). Rep Dir Vet Res.

[4] Besier B (2007). New anthelmintics for livestock: the time is right. Trends Parasitol.

[5] Jabbar A, Iqbal Z, Kerboeuf D, Muhammad G, Khan MN, Afaq M (2006). Anthelmintic resistance: The state of play revisited. Life Sci.

[6] Kaplan R, Vidyashankar A (2012). An inconvenient truth: global worming and anthelmintic resistance. Vet Parasitol..

[7] Kaplan RM (2004). Drug resistance in nematodes of veterinary importance: A status report. Trends Parasitol.

[8] Scott I, Pomroy WE, Kenyon PR, Smith G, Adlington B, Moss A (2013). Lack of efficacy of monepantel against *Teladorsagia circumcincta* and *Trichostrongylus colubriformis*. Vet Parasitol.

[9] Wolstenholme AJ, Fairweather I, Prichard R, Von Samson-Himmelstjerna G, Sangster NC (2004). Drug resistance in veterinary helminths. Trends Parasitol.

[10] Wolstenholme AJ, Kaplan RM (2012). Resistance to macrocyclic lactones. Curr Pharm Biotechnol.

[11] Kaminsky R, Ducray P, Jung M, Clover R, Rufener L, Bouvier J, Weber SS, Wenger A, Wieland-Berghausen S, Goebel T. A new class of anthelmintics effective against drug-resistant nematodes. Nature. 2008;452(7184):176–80. 10.1038/nature06722 18337814

[12] Prichard RK, Geary TG (2008). Drug discovery: Fresh hope to can the worms. Nature.

[13] Lee BH, Clothier MF, Johnson SS (2001). Semi-synthesis of 2-deoxo- and 3-epi-paraherquamide A. Bioorg Med Chem Lett.

[14] Hammond JA, Fielding D, Bishop SC (1997). Prospects for plant anthelmintics in tropical veterinary medicine. Vet Res Commun.

[15] Waller PJ, Bernes G, Thamsborg SM, Sukura A, Richter SH, Ingebrigtsen K, Höglund J.. Plants as de-worming agents of livestock in the Nordic countries: historical perspective, popular beliefs and prospects for the future. Acta Vet Scand. 2001;42(1):31–44. 10.1186/1751-0147-42-31 PMC2202332 11455900

[16] Liu ZL, Liu JP, Zhang AL, Wu Q, Ruan Y, Lewith G, Visconte D. Chinese herbal medicines for hypercholesterolemia. Cochrane Database Syst Rev. 2011;7:CD008305. 10.1002/14651858.CD008305.pub2 PMC3402023 21735427

[17] Xu HB, Jiang RH, Chen XZ, Li L (2012). Chinese herbal medicine in treatment of diabetic peripheral neuropathy: A systematic review and meta-analysis. J Ethnopharmacol.

[18] Hon KLE, Ma KC, Wong Y, Leung TF, Fok TF (2005). A survey of traditional Chinese medicine use in children with atopic dermatitis attending a paediatric dermatology clinic. J Dermatol Treat.

[19] Li ZH, Wan JY, Wang GQ, Zhao FG, Wen JH (2013). Identification of compounds from *Paris polyphylla* (ChongLou) active against *Dactylogyrus intermedius*. Parasitology.

[20] Zhu L, Dai JL, Yang L, Qiu J (2013). *In vitro* ovicidal and larvicidal activity of the essential oil of *Artemisia lancea* against *Haemonchus contortus* (Strongylida). Vet Parasitol.

[21] Hrckova G, Velebny S (2013). Parasitic helminths of humans and animals: health impact and control. Pharmacological Potential of Selected Natural Compounds in the Control of Parasitic Diseases.

[22] Roeber F, Kahn L (2014). The specific diagnosis of gastrointestinal nematode infections in livestock: Larval culture technique, its limitations and alternative DNA-based approaches. Vet Parasitol..

[23] Eguale T, Tadesse D, Giday M (2011). *In vitro* anthelmintic activity of crude extracts of five medicinal plants against egg-hatching and larval development of *Haemonchus contortus*. J Ethnopharmacol.

[24] Hoste H, Jackson F, Athanasiadou S, Thamsborg SM, Hoskin SO (2006). The effects of tannin-rich plants on parasitic nematodes in ruminants. Trends Parasitol.

[25] Kaewintajuk K, Cho PY, Kim SY, Lee ES, Lee HK, Choi EB, Park H. Anthelmintic activity of KSI-4088 against *Caenorhabditis elegans*. Parasitol Res. 2010;107(1):27–30. 10.1007/s00436-010-1828-8 20309581

[26] Kumarasingha R, Palombo EA, Bhave M, Yeo TC, Lim DSL, Tu CL, Shaw JM, Boag PR. Enhancing a search for traditional medicinal plants with anthelmintic action by using wild type and stress reporter *Caenorhabditis elegans* strains as screening tools. Int J Parasitol. 2014;44(5):291–8. 10.1016/j.ijpara.2014.01.008 24583111

[27] Chai PPK. Medicinal Plants of Sarawak: Paul Chai P.K.; 2006.

[28] Christensen H (2002). Ethnobotany of the Iban & the Kelabit: Nepcon.

[29] Preston S, Jabbar A, Nowell C, Joachim A, Ruttkowski B, Baell J, Cardno T, Korhonen PK, Piedrafita D, Ansell BRE. Low cost whole-organism screening of compounds for anthelmintic activity. Int J Parasitol. 2015;45(5):333–43. 10.1016/j.ijpara.2015.01.007 25746136

[30] Preston S, Jabbar A, Nowell C, Joachim A, Ruttkowski B, Cardno T, Hofmann A, Gasser RB. Practical and low cost whole-organism motility assay: A step-by-step protocol. Mol Cell Probes. 2015. doi:10.1016/j.mcp.2015.08.005. 10.1016/j.mcp.2015.08.005 26365227

[31] Schwarz E, Korhonen P, Campbell B, Young N, Jex A, Jabbar A, Hall R, Mondal A, Howe A, Pell J et al. The genome and developmental transcriptome of the strongylid nematode *Haemonchus contortus*. Genome Biol. 2013;14(8):R89. 10.1186/gb-2013-14-8-r89 PMC4053716 23985341

[32] Nikolaou S, Hartman D, Presidente P, Newton S, Gasser RB (2002). HcSTK, a *Caenorhabditis elegans* PAR-1 homologue from the parasitic nematode, *Haemonchus contortus*. Int J Parasitol..

[33] Sommerville RI (1966). The Development of *Haemonchus contortus* to the fourth stage *in vitro*. J Parasitol.

[34] Maizels RM, Blaxter ML, Selkirk ME (1993). Forms and functions of nematode surfaces. Exp Parasitol.

[35] Ríos-de álvarez L, Jackson F, Greer A, Bartley Y, Bartley DJ, Grant G, Huntley JF. *In vitro* screening of plant lectins and tropical plant extracts for anthelmintic properties. Vet Parasitol. 2012;186(3–4):390–8. 10.1016/j.vetpar.2011.11.004 22130336

[36] Rudin W (1990). Comparison of the cuticular structure of parasitic nematodes recognized by immunocytochemical and lectin binding studies. Acta Trop.

[37] Sager H, Bapst B, Strehlau GA, Kaminsky R (2012). Efficacy of monepantel, derquantel and abamectin against adult stages of a multi-resistant *Haemonchus contortus* isolate. Parasitol Res.

[38] Blaxter ML. Nematodes: The worm and its relatives. PloS Biol. 2011;9(4):e1001050. 10.1371/journal.pbio.1001050 PMC3079589 21526226

[39] Geary TG, Thompson DP (2001). *Caenorhabditis elegans*: How good a model for veterinary parasites?. Vet Parasitol.

[40] Laing R, Kikuchi T, Martinelli A, Tsai IJ, Beech RN, Redman E, Holroyd N, Bartley DJ, Beasley H, Britton C et al. The genome and transcriptome of *Haemonchus contortus*, a key model parasite for drug and vaccine discovery. Genome Biol. 2013; 14(8):R88. 10.1186/gb-2013-14-8-r88 PMC4054779 23985316

[41] Burns AR, Wallace IM, Wildenhain J, Tyers M, Giaever G, Bader GD, Nislow C, Cutler SR, Roy PJ. A predictive model for drug bioaccumulation and bioactivity in *Caenorhabditis elegans*. Nature Chem Biol. 2010;6(7):549–57. 10.1038/nchembio.380 20512140

